# What is a ‘timely’ diagnosis? Exploring the preferences of Australian health service consumers regarding when a diagnosis of dementia should be disclosed

**DOI:** 10.1186/s12913-018-3409-y

**Published:** 2018-08-06

**Authors:** Rochelle Watson, Jamie Bryant, Robert Sanson-Fisher, Elise Mansfield, Tiffany-Jane Evans

**Affiliations:** 1 Health Behaviour Research Collaborative, School of Medicine and Public Health, Faculty of Health and Medicine, University of Newcastle, W4 HMRI Building, Callaghan, NSW 2308 Australia; 2 Priority Research Centre for Health Behaviour, University of Newcastle, Callaghan, NSW Australia; 3 Hunter Medical Research Institute, Lot 1 Kookaburra Cct, New Lambton Heights, NSW 2305 Australia

**Keywords:** Dementia, Diagnosis disclosure, Timely diagnosis, Consumer preferences

## Abstract

**Background:**

Recently the dementia field has shifted focus away from the early diagnosis debate in favour of ‘timely’ diagnosis. ‘Timely’ diagnosis disclosure takes into consideration the preferences and unique circumstances of the individual. Determining when diagnosis disclosure is ‘timely’ may be particularly complex if there are differing views between the individual, their family, and their health care providers regarding disclosure. This study explores the preferences of consumers regarding when a diagnosis of dementia should be communicated.

**Methods:**

A cross-sectional survey was conducted with English-speaking adults attending outpatient clinics at an Australian hospital. Participants were recruited by a research assistant in the clinic waiting room and invited to complete the survey on a web-connected iPad. The survey included questions examining socio-demographics and experience with dementia. Two scenarios were used to explore preferences for timing of diagnosis disclosure.

**Results:**

Of 446 participants, 92% preferred a diagnosis of dementia to be disclosed as soon as possible. Preferences were not associated with socio-demographics or previous dementia experience. Most participants also preferred disclosure to occur as soon as possible if their spouse or partner was diagnosed with dementia (88%). There was strong correlation between preferences for self and preferences for spouse (0.91).

**Conclusions:**

These findings provide guidance to health care providers about preferences for disclosure of a dementia diagnosis, and may help to overcome potential barriers to timely diagnosis. As the prevalence of dementia increases, consumers’ preference for diagnosis to occur as soon as possible has important implications for the health system.

**Electronic supplementary material:**

The online version of this article (10.1186/s12913-018-3409-y) contains supplementary material, which is available to authorized users.

## Background

### The benefits and risks of early diagnosis of dementia

Diagnosis of dementia often occurs as a result of a crisis event that causes disruption to a previously stable or well-managed situation [1]. Early diagnosis of dementia typically refers to the detection of the first signs and symptoms of dementia prior to such an event occurring [2]. While research suggests that a diagnosis of dementia is often a cumulative process that occurs over time, rather than as a discrete act at a particular point in time [2], there has been significant debate in the field of dementia regarding the benefit of early diagnosis and implications of diagnosis disclosure which occurs early on in the dementia trajectory [3]. Reported benefits of early diagnosis of dementia include: enabling early implementation of interventions to maintain independence for as long as possible; providing time for the person with dementia to make decisions about future financial, legal, and medical issues while they have capacity; and providing more time to establish contact with support services and networks [3]. However, given there is no treatment available to cure or significantly slow the progression of dementia, the position of some health professionals is that early diagnosis of dementia is irrelevant and even detrimental to individuals’ emotional wellbeing [4, 5]. The potential for false positives and the resulting strain this may have on already limited resources, is also of concern [2].

### Timely diagnosis disclosure reflects person-centred care

In recent years, there has been a shift away from debating the relative benefit of early diagnosis in favour of timely diagnosis, reflecting the person-centred care movement [2]. Person-centred care refers to care that is responsive to consumer needs, values and preferences; allows for the involvement of family and friends; and supports the provision of information, communication and education to enable consumers to understand and make informed decisions about their care [6]. Person-centred care should occur across the dementia trajectory, from diagnosis to end-of-life, however this often relies on the person receiving a timely and supported diagnosis [7]. The concept of timely diagnosis refers to disclosure of the diagnosis at the right time for the individual with consideration of their preferences and unique circumstances [2]. Where early diagnosis disclosure assumes all people with dementia want to be told their diagnosis as soon as possible, timely diagnosis disclosure treats people with dementia as individuals with unique perspectives. Given the highly individualised nature of timely diagnosis, the right time for diagnosis disclosure may be very different for different people. For example, some people may wish to be told their diagnosis as soon as clinical tools indicate a probable diagnosis of dementia, while others might prefer not to be told they have dementia at all. Both scenarios may represent timely diagnosis disclosure according to the perceptions of the consumer.

### Determining when diagnosis disclosure is considered ‘timely’

Determining when diagnosis disclosure is timely may be particularly complex if there are differing views between the consumer, their family, and their health care providers about the most appropriate time to discuss a diagnosis of dementia. Across a range of life-threatening illnesses, health care providers do not often accurately predict their patient’s preferences, particularly in relation to involvement in decision making about their care [8, 9] or end of life care [10]. Perceptions of timely diagnosis disclosure may also differ when considering preferences for one’s own care versus preferences for the care of a loved one. Previous studies have found many relatives or carers of people with dementia do not want their loved one to be told their diagnosis, despite indicating they would want to be told if they themselves had dementia [11]. Such discordance in views has the potential to cause conflict among family members and may further contribute to the complexity of determining timely diagnosis disclosure. This emphasises the need for clinicians to take a person-centred approach, by asking each individual consumer about their preferences for dementia diagnosis disclosure, rather than relying on potentially incorrect assumptions or decisions made on the person’s behalf. Australian clinical practice guidelines for dementia recommend that ‘people have the right to know their diagnosis and the right not to know their diagnosis’ [12].

### Addressing gaps in the literature

While numerous studies have explored community and carer perceptions of whether or not a diagnosis of dementia should be disclosed to the individual with the condition [11, 13], a 2013 review of the literature found no Australian studies examining consumer views [14]. Previous research also does not clearly address the issue of timing of disclosure. This gap in knowledge is likely due to the relatively recent emergence of the concept of timely diagnosis. Given the emphasis and support for this concept by stakeholders and policy makers in Australia [15], there is a need to explore the preferences of consumers regarding, not just *if* but, *when* a diagnosis of dementia should be communicated.

This study examined among a sample of health service consumers:

- Aim 1: Preferences regarding the timing of diagnosis disclosure for dementia in relation to a) themselves, and b) their spouse;
- Aim 2: Whether socio-demographic characteristics and previous experience with dementia are associated with preferences for diagnosis; and.
- Aim 3: Agreement between preferences regarding timing of diagnosis disclosure for dementia for self versus preferences for spouse.

## Methods

### Design & setting

A cross-sectional survey of health care consumers attending outpatient clinics (see Additional file 1) within one large, regional Australian tertiary referral hospital was conducted. Data was collected over a two-month period in 2016–2017. This study was approved by the Hunter New England Human Research Ethics Committee, and registered with the University of Newcastle Human Research Ethics Committee.

### Participants

Eligible participants were aged 18 years or older; attending an outpatient appointment at the participating hospital, either as a patient or person accompanying a patient; had sufficient English to complete the survey; and judged to be mentally and physically well enough to complete an iPad survey. Younger as well as older participants were included, as younger adults are also likely to be involved in decision making about whether loved ones should be assessed for dementia (e.g. their parents). Participants who indicated they had received a diagnosis of dementia or had completed the survey at a previous appointment were excluded.

### Recruitment

Trained research assistants approached potential participants in the outpatient clinic waiting rooms to introduce the study and provide a printed information sheet. Research assistants checked eligibility and provided willing participants with an introduction to the iPad touch screen technology, where required. Participants completed a consent form electronically via the iPad. The age and gender of non-consenters was recorded by research assistants to enable examination of consent bias.

### Data collection

Consenting participants were asked to complete an anonymous online survey on a web-connected iPad while waiting for their appointment. Research assistants provided participants with assistance using the iPad where requested. Participants who were called in for their appointment prior to finishing the survey were invited to continue with the survey after their appointment if they wished to. A unique identifier was used to allow participants to save responses and enable completion of the survey after their appointment. QuON software was used to program and administer the survey via a secure server [16]. QuON is a web-based survey software application that allows for sophisticated branching of questions using pre-programmed algorithms, to ensure survey questions are tailored based on the participant’s previous responses [16].

### Measures

Development of the survey involved an iterative process of review and refinement, including: 1) Critical appraisal of the relevant literature examining the advantages and disadvantages of early diagnosis of dementia; 2) A small focus group with six members of the community aged over 65 years with no previous diagnosis of dementia to expand on data from Step 1, and explore perceived advantages and/disadvantages of early diagnosis; 3) Development of survey items and peer review by four public health researchers; and 4) Pilot testing of the final draft survey with an initial 20 participants (recruited as per the methods previously described), with further refinements made as necessary.

The survey questions (see Additional file 2) were embedded within a larger survey which also included questions about health-related decision making. The larger survey took approximately 10 min to complete. The following items are reported here:

#### Socio-demographics

Participants self-reported their age, gender, education, employment status, and relationship status.

#### Experience with dementia

Participants were asked if they themselves had previously been diagnosed with dementia, whether they know someone with dementia, and if so, their relationship to the person.

#### Preferences for timing of diagnosis disclosure

A lay description of dementia was provided: “*Dementia (sometimes called ‘Alzheimer’s’) affects thinking, behaviour and the ability to perform everyday tasks. There is currently no cure or treatment to reverse or stop the progression of dementia.*” Following this, two scenarios were presented to elicit participants’ preferences for receiving a diagnosis of dementia. Participants were first asked ‘*Imagine you see your doctor and test results show that you have dementia. Given there is no cure, when would you want your doctor to tell you that you have dementia?’*. Participants were asked to select one of four response options available for selection (1 = *As soon as possible, 2 = Not until my symptoms got worse or made me really worried, 3 = Only when my family thought it was necessary to tell me, 4 = I would not want to be told at all*). Participants were then asked to indicate the reasons for their preference. The list of reasons presented were tailored depending on the response selected for the scenario. Participants were able to select as many reasons as applied. Participants who selected response option 1 were presented with reasons associated with advantages of early diagnosis (e.g. *So I could have more time to be involved in decisions about my future care*), while participants who selected response options 2–4 were presented with reasons associated with disadvantages of early diagnosis (e.g. *It would cause me to feel depressed*). Participants also had the option of entering their own reasons into the survey. Participants who indicated they had a spouse or partner who did not have a diagnosis of dementia were then presented with the second hypothetical scenario which asked ‘*Imagine your partner/spouse sees their doctor and test results show that they have dementia. Given there is no cure, when would you want to them to be told that they have dementia?’*. The same response options and follow up questions were presented as described above (with appropriate wording changes).

### Statistical analysis

Statistical analyses were conducted using IBM SPSS Statistics v23 [17] and SAS software v9.4 [18]. Consent bias was examined using chi-square tests to compare age and gender of non-consenters versus participants. Data from the iPad survey was analysed using descriptive statistics (percentages and 95% confidence intervals for the true proportion) to examine preferences regarding the timing of diagnosis disclosure for dementia and reasons for these (Aim 1). Given low cell counts within the preference for disclosure variable, response options regarding preferences for timing of diagnosis disclosure were collapsed into two groups: 1) as soon as possible; and 2) later or no disclosure, which incorporated all other response options. An exact logistic regression was conducted with the binary outcome (as soon as possible vs. later or no disclosure) to explore whether socio-demographic characteristics and previous experience with dementia were associated with preferences for diagnosis disclosure (Aim 2). The following variables were included in the regression as possible predictors: age, gender, education, employment status, and experience with dementia. Agreement between preferences for the self versus preferences for their spouse was examined using a test of tetrachoric correlation (Aim 3).

## Results

### Sample characteristics

Seventy-seven percent (*N* = 463) of the 601 potential participants identified as eligible consented to participate in the study. Of these, survey data was available for 448 participants; two were subsequently excluded as they had been previously diagnosed with dementia. Comparison of gender between non-consenters and participants revealed no indication of consent bias (χ^2^ = 0.421, *p* = 0.517). The majority of participants were female (57%) and reported having a spouse or partner (74%). Forty-one percent of participants were aged 18–49 years, 57% aged 50–59 years, 32% aged 60–74 years, and 10% were aged 75 years or older (see Table 1). Participants were primarily attending for orthopaedics (*N* = 114; 25.6%), neurosurgery and neurology (*N* = 55; 12.4%) and ear, nose, throat & eye (*N* = 45; 10.1%) outpatient appointments (see Additional file 1 for further detail).

**Table 1:** Participant demographic characteristics (*n* = 446)^a^

Characteristic		*N* (%)
Gender	Male	181 (40.6%)
	Female	254 (57%)
Age	18–49	141 (31.6%)
	50–59	108 (24.2%)
	60–74	141 (31.6%)
	75+	44 (9.9%)
Highest level of Education	High school or below	168 (37.7%)
	Trade or vocational training (e.g. TAFE or college)	130 (29.1%)
	University or post-graduate degree	72 (16.1%)
	Other	7 (1.6%)
Employment status	Full-time employment	91 (20.4%)
	Part-time or casual employment	56 (12.6%)
	Unemployed	19 (4.3%)
	Disability pension	35 (7.8%)
	Retired	114 (25.6%)
	Home duties	21 (4.7%)
	Student	7 (1.6%)
	Other	34 (7.6%)
Relationship Status	Married or living with partner	246 (55.2%)
	Divorced or separated	43 (9.6%)
	Widowed	22 (4.9%)
	Never married	59 (13.2%)

^a^Columns do not add to 446 due to missing data

### Experience with dementia

Almost half of all participants (*N* = 189, 46%; 95%CI 41–51) reported knowing someone with dementia. The majority of these participants reported having a friend (*N* = 68, 36%), parent (*N* = 59, 31%; including in-laws), or grandparent (*N* = 21, 11%; including in-laws) with the condition.

### Personal preferences for dementia diagnosis disclosure

The majority of participants preferred to find out if they had dementia as soon as possible (*N* = 375, 92%; 95%CI 89–94). Two percent (*N* = 9; 95%CI 0.7–3.7) indicated they would not want to know until symptoms got worse, 3% (*N* = 12; 95%CI 1.5–4.7) indicated they would only want to be told when their family thought it was necessary, and 3% (*N* = 12; 95%CI 1.5–4.7) preferred to not find out the diagnosis at all. Reasons for preferring to know the diagnosis as soon as possible are presented in Table 2. The most frequently reported reason was to ‘make the most of life’ (*N* = 277, 75%). For participants who indicated ‘Other’ reasons (*N* = 13, 4%), these included: wanting to take control and find alternative treatments or natural therapies, brain retraining and trying to delay the disease, and wanting to know everything. Where a later or no disclosure was preferred (*N* = 33, 8%), the most commonly reported reason was ‘to avoid unnecessary worry’ (*N* = 20, 61%). Reasons for preferring to know the diagnosis later or not at all are presented in Table 3.

**Table 2:** Reasons for a preference for diagnosis disclosure ‘as soon as possible’

Reasons	Self (*n* = 371^a^)	Spouse (*n* = 258^b^)
	N (%)	N (%)
Make the most of life (e.g. ‘bucket list’)	277 (75%)	189 (73%)
Be involved in decisions about future care	265 (71%)	196 (76%)
Access treatments and support	253 (68%)	183 (71%)
Time to come to terms with diagnosis	253 (68%)	187 (73%)
Make financial arrangements	239 (64%)	169 (66%)
Tell loved ones my situation	229 (62%)	163 (63%)
Collect memories	170 (46%)	132 (51%)
Time to work on relationships	134 (36%)	108 (42%)
Other	13 (4%)	8 (3%)

^a^Missing data N = 4

^b^Missing data N = 2

**Table 3:** Reasons for a preference for diagnosis disclosure ‘later or no disclosure’

Reasons	Self (*n* = 33)	Spouse (*n* = 33^a^)
	N (%)	N (%)
To avoid unnecessary worry	20 (61%)	18 (55%)
There is no cure / no benefit of knowing	17 (52%)	23 (70%)
Fear that people would treat me/them differently	12 (36%)	11 (33%)
It would cause me/them to feel depressed	11 (33%)	18 (55%)
To avoid putting strain on relationships	9 (27%)	10 (30%)
Symptoms might progress faster	6 (18%)	7 (21%)
Risk of incorrect diagnosis	3 (9%)	5 (15%)
To live normally for as long as possible	2 (6%)	14 (42%)

^a^Missing data N = 2

### Association between disclosure preferences and socio-demographic characteristics

Participants with complete data for all analysis variables were included in the regression (*N* = 377). Table 4 shows the odds ratios, 95% confidence intervals and *p*-values from the exact logistic regression. Preferences regarding timing of diagnosis disclosure were not significantly associated with previous dementia experience or any of the socio-demographics examined.

**Table 4:** Association between explanatory variables and preference for diagnosis disclosure

Explanatory Variable	Total^a^ (*N* = 377)	ASAP^a^ (*n* = 345)	Later Never^a^ (*n* = 32)	OR (95% CI)	*P* Value
Gender					
Female	220 (58)	202 (59)	18 (56)	1.14 (0.49–2.61)	0.876
Male	157 (42)	143 (41)	14 (44)	ref	–
Age					
18–49 Years	125 (33)	118 (34)	7 (22)	1.40 (0.52–4.22)	0.632
> 50 Years	252 (67)	227 (66)	25 (78)	ref	–
Education					
≤ High School	173 (46)	156 (45)	17 (53)	0.90 (0.39–2.05)	0.926
Trade or Tertiary	204 (54)	189 (55)	15 (47)	ref	–
Employment					
Paid Work	154 (41)	146 (42)	8 (25)	1.89 (0.73–5.40)	0.227
Unpaid Work/Pension/Unemployed	223 (59)	199 (58)	24 (75)	ref	–
Experience with dementia					
Yes	169 (45)	154 (45)	15 (47)	0.88 (0.38–2.03)	0.890
No	208 (55)	191 (55)	17 (53)	ref	–

^a^Figures in parentheses are percentages

### Preferences for dementia diagnosis disclosure for spouse/partner

The majority of participants also preferred diagnosis disclosure to occur as soon as possible for their spouse or partner (*N* = 260, 88%; 95%CI 84–92). Four percent (*N* = 12; 95%CI 2–6.8) indicated they would not want their spouse to know until the symptoms got worse, 6% (*N* = 17; 95%CI 3.4–8.5) indicated they would only want their spouse to be told when the family thought it was necessary, and 2% (*N* = 6; 95%CI 0.7–3.7) preferred for their spouse not to be told the diagnosis at all. Reasons for preferring disclosure as soon as possible are presented in Table 2. The most frequently reported reason was so their spouse could be involved in decisions about their future care (*N* = 196, 76%). Where a later or no diagnosis was preferred (*N* = 35, 12%), 70% (*N* = 23) of participants indicated ‘there is no cure/ no benefit of them knowing’ as the reason for this preference. Reasons for preferring disclosure later or not at all are presented in Table 3.

### Agreement between preferences for self versus preferences for spouse

There was a strong correlation between preferences for self and spouse regarding timing of diagnosis disclosure (0.91, 95%CI 0.84 to 0.99). Participants who indicated a preference to be told their diagnosis of dementia ‘as soon as possible’ had 76.5 times higher odds of choosing ‘as soon as possible’ for their spouse, compared to those who preferred diagnosis disclosure to occur later or not at all.

## Discussion

An overwhelming majority (92%) of health service consumers wanted to be told their diagnosis as soon as possible if they had dementia. Despite variation in the socio-demographic characteristics of participants and previous experience with dementia, these factors did not significantly impact preferences for timing of diagnosis disclosure. Our finding is consistent with previous research that also shows that most people want to be told their diagnosis. For example, a survey conducted with medical outpatients in the United States found that 92% of participants wanted to be told if they had dementia [19], and 96% of outpatients attending a memory clinic in the Netherlands, and their accompanying relatives, thought it was important to be told a diagnosis of dementia [20]. However, these previous studies did not explore *when* the diagnosis should be disclosed. The findings of the current study indicate that the majority of this group of health service consumers would like to know their diagnosis as early as possible. This finding may reflect assertion of ‘the right to know their diagnosis’ [12] or increased confidence among the Australian community that there is appropriate support and interventions available for people with dementia, potentially as a result of the widely-promoted research investment in dementia [21] and media presence of dementia advocacy organisations, such as Alzheimer’s Australia [22], in recent years. Importantly, this study provides new information about the reasons why health service consumers would like to know their diagnosis as soon as possible, with reasons similar for both the participant themselves, and their spouse/partner. This information may assist health care providers to understand the values and concerns of consumers and their families in relation to diagnosis disclosure and tailor their care accordingly.

When considering diagnosis disclosure for a spouse or partner, participants also overwhelmingly preferred disclosure to occur as soon as possible (88%). This finding is also consistent with previous research exploring whether outpatients would want their spouse to be given a diagnosis if the spouse had dementia [19]. The strong correlation between preferences for self and spouse regarding timing of diagnosis disclosure may suggest that in most cases, spouses would be supportive of the individual’s decision regarding timing of diagnosis disclosure. However, in the current study, participants were only asked about preferences for dementia diagnosis if they or their spouse did not have any previous history of dementia. Interestingly, previous research that has explored diagnosis disclosure with carers or relatives of people with dementia have found that preferences for disclosure are extremely varied [11, 14], ranging from as few as 17% of carers in one study [23] to 97% in another [24]. This suggests that the experience of supporting a relative to cope with the symptoms of dementia and experience of obtaining a diagnosis may influence caregiver’s perceptions about the perceived benefits and harms of diagnosis disclosure to the person with dementia. It also highlights the potential limitations of the use of a hypothetical scenario to elicit preferences for diagnosis disclosure, which may not accurately reflect preferences when faced with these circumstance in real life. Future research could explore whether people with dementia and their carers would have wanted to find out the diagnosis earlier or later given the benefit of hindsight.

The results of the current study demonstrate the wide-reaching impact of dementia on the community, with 46% of participants indicating that they knew someone with dementia. Almost a third of these had a parent or parent in-law with the condition. Considering the incidence of dementia in Australia is predicted to increase from 244 people diagnosed each day in 2017 to 451 per day by 2036 [25], the significant proportion of people whose lives are impacted by dementia is likely to also increase.

### Limitations

Some limitations of the study methodology should be considered when interpreting study findings. Previous Australian research has found that willingness to be tested for Alzheimer’s disease varied depending on the type and invasiveness of testing [26]. The scenario employed in the current study was framed from a point in time after the individual had seen their doctor and had tests done which indicated dementia. The authors acknowledge that this scenario may oversimplify the assessment and diagnostic processes involved in determining a dementia diagnosis, and could have impacted on preferences regarding the *timing* of diagnosis disclosure. Investigation of associations between socio-demographic characteristics and preferences was also limited by the lack of variation in participants’ preferences for diagnosis disclosure. Additionally, the response options for the questions asking participants about the reasons for their preference regarding timing of diagnosis were tailored depending on the response selected for the scenario. Presenting participants with a comprehensive list of reasons associated with advantages of early diagnosis and reasons associated with disadvantages of early diagnosis could have resulted in different responses. There was a need to dichotomise the explanatory variables for the regression analysis, which may have limited the sensitivity of this test. Given the sample was recruited from a regional hospital and inclusion criteria was limited to English speaking participants, the views of consumers from culturally and linguistically diverse backgrounds may not be well-represented. It is possible that a more culturally heterogeneous sample recruited from a metropolitan setting may find greater variation in preferences for timing of disclosure.

### Implications

The current study adds to our understanding of the concept of a timely diagnosis, and provides valuable insight into the preferences of Australian health care consumers regarding when a dementia diagnosis should be disclosed. This information can provide guidance to health care providers about what most people might want if a diagnosis of dementia was probable, and may help to overcome potential barriers to timely diagnosis associated with negative provider views on disclosure [27, 28]. However, it is important to note that such information cannot replace the essential processes of person-centred care, particularly given the potential for the experience of living with the symptoms of early stage dementia to change preferences for care. People with cognitive impairment are capable of expressing their preferences in relation to their health care and desire for involvement in decision making [29, 30]. All consumers should be given the opportunity to indicate their preferences about if and when they would like to be told about a diagnosis of dementia. Enabling people with dementia to exercise such control may be the first important step in ensuring enduring person-centred care and respect for autonomy from pre-diagnosis to later life.

Findings of the current study may also have broader implications for the health system. The prevalence of dementia in Australia is expected to increase by 90% in the next 20 years [25]. Results of the current study indicate that a significant majority of those who will be faced with a diagnosis of dementia would want to be told their diagnosis as early as possible. Consumer preferences for an earlier diagnosis may lead to increased demand for dementia assessment and support services. Given the existing shortage of specialists in dementia care, particularly in regional and rural areas [31], the increased demand is likely to lead to greater reliance on the primary care sector. Barriers to timely diagnosis of dementia and disclosure in primary care have been reported, including a lack of confidence, skills, and time [28]. This highlights the potential need for improved dementia-specific training for primary care physicians [28]. There may also be value in extending the role of practice nurses to include dementia assessment and management [28]. Integrating practice nurses into dementia care in this way may serve to ease the burden on primary care physicians, while also providing people with dementia and their caregivers with greater continuity of care and accessible support throughout the dementia trajectory.

## Conclusions

The majority of health consumers preferred for diagnosis disclosure to occur as soon as possible if they or their spouse had dementia. Preferences were not influenced by socio-demographic characteristics or previous experience with dementia. A timely diagnosis is likely to be highly individualised and specific to the unique circumstances of the individual at a particular point in time. Providing every person with the opportunity to express their preferences for not only if but when disclosure of a dementia diagnosis should occur, is essential for ensuring care is person-centred.

## Additional files

Additional file 1: Distribution of participants among outpatient clinics. (DOCX 12 kb)

Additional file 2: Survey items. (DOCX 25 kb)
