# Supplementary material for: What is a ‘timely’ diagnosis? Exploring the preferences of Australian health service consumers regarding when a diagnosis of dementia should be disclosed
Source: BMC Health Serv Res. 2018 Aug 6;18:612. doi: 10.1186/s12913-018-3409-y (PMC6080387; doi:10.1186/s12913-018-3409-y)
Supplement: Supplementary file 1 — Distribution of participants among outpatient clinics. (DOCX 12 kb) [file 12913_2018_3409_MOESM1_ESM.docx]

**Distribution of participants among outpatient clinics**

| **Outpatient Clinic** | **N** | **%** |
| --- | --- | --- |
| Orthopaedics | 114 | 25.6% |
| Neurosurgery and Neurology | 55 | 12.4% |
| Ear, Nose, Throat & Eye | 45 | 10.1% |
| Other | 45 | 10.1% |
| General surgery | 25 | 5.6% |
| Gastroenterology | 23 | 5.2% |
| Respiratory | 20 | 4.5% |
| Immunology | 18 | 4.0% |
| Cardiology | 17 | 3.8% |
| Urology | 17 | 3.8% |
| Vascular surgery | 13 | 2.9% |
| Rheumatology | 11 | 2.5% |
| Endocrinology | 11 | 2.5% |
| Missing | 11 | 2.5% |
| Not Sure | 9 | 2.0% |
| Dermatology | 7 | 1.6% |
| Renal | 5 | 1.1% |
