# Supplementary material for: What is a ‘timely’ diagnosis? Exploring the preferences of Australian health service consumers regarding when a diagnosis of dementia should be disclosed
Source: BMC Health Serv Res. 2018 Aug 6;18:612. doi: 10.1186/s12913-018-3409-y (PMC6080387; doi:10.1186/s12913-018-3409-y)
Supplement: Supplementary file 2 — Survey items. (DOCX 25 kb) [file 12913_2018_3409_MOESM2_ESM.docx]

**Survey Items**

**MODULE 1**

**We will use the following information to ensure you are only asked questions that are relevant to your gender and age.**

| 1. **What is your age?** | □ 18-49 years  □ 50-59 years  □ 60-74 years  □ Over 75 years |  |
| --- | --- | --- |
| 1. **Are you?** | □ Male  □ Female | |
| 1. **What is the highest level of schooling you have completed? *Please select one answer only*.** | □ High school or below  □ Trade or vocational training (e.g. TAFE or college)  □ University of postgraduate degree  □ Other – please specify ______________________ | |
| 1. **How would you best describe your employment situation at the moment?** | □ Employed full time  □ Employed part time/casual  □ Unemployed  □ Disability pension  □ Retired  □ Home duties  □ Student  □ Other – please specify ______________________ | |
| 1. **What is your current marital status?** | □ Married or living with partner  □ Divorced or separated  □ Widowed  □ Never married | |

**MODULE 3**

**Dementia (sometimes called 'Alzheimers') affects thinking, behaviour and the ability to perform everyday tasks.**

**There is currently no cure or treatment to reverse or stop the progression of dementia.**

| 1. **Have you been diagnosed with dementia by a health care professional?** | □ Yes 🡪 skip to next section  □ No | |
| --- | --- | --- |
| 1. **Do you know someone with dementia?** | □ Yes  □ No |  |
| 1. **What is your relationship to this person?** | *S/he is my:*  □ Partner/spouse  □ Friend  □ Parent  □ Other (please specify) | |

**The next questions ask about your preferences for receiving a diagnosis of dementia.**

**These questions are hypothetical (imaginary).**

**We do not know anything about your current health.**

| ***Scenario 1***  *Please remember this situation is imaginary.*  *Imagine you see your doctor and test results show that you have dementia.*  **Given there is no cure, when would you want your doctor to tell you that you have dementia?** |
| --- |
| 🗆 As soon as possible  🗆 I would not want to know until my symptoms got worse or made me really worried  🗆 Only when my family thought it was necessary to tell me  🗆 I would not want to know my diagnosis at all |

**We would like to know more about why you chose this option.**

| **5a. Please select all the reasons that apply from the list below.** |
| --- |
| *So I could have more time to:*  🗆 Make the most of life (e.g. ‘bucket list’)  🗆 Collect memories  🗆 Work on my relationships  🗆 Tell loved ones about my situation  🗆 Come to terms with the diagnosis  🗆 Be involved in decisions about my future care  🗆 Make financial arrangements for with family  🗆 Access treatments and support  🗆 Other (please specify) |

**We would like to know more about why you chose this option.**

| **5b. Please select all the reasons that apply from the list below.** |
| --- |
| 🗆 There is no cure / no benefit of knowing  🗆 Risk of incorrect diagnosis  🗆 It would cause me to feel depressed  🗆 Fear that people would treat me differently  🗆 To avoid unnecessary worry  🗆 To avoid putting strain on my relationships  🗆 Fear that knowing the diagnosis might make symptoms progress faster  🗆 So I could live normally for as long as possible  🗆 Other (please specify) |

| ***Scenario 2***  *Please remember this situation is imaginary.*  *Imagine your partner or spouse sees their doctor and test results show that they have dementia.*  **Given there is no cure, when would you want them to be told that they have dementia?** |
| --- |
| 🗆 As soon as possible  🗆 Not until their symptoms got worse or made them really worried  🗆 Only when the family thought it was necessary to tell them  🗆 I would not want them to be told at all |

**We would like to know more about why you chose this option.**

| **7a. Please select all the reasons that apply from the list below.** |
| --- |
| *So they could have more time to:*  🗆 Make the most of life (e.g. ‘bucket list’)  🗆 Collect memories  🗆 Work on their relationships  🗆 Tell loved ones about their situation  🗆 Come to terms with the diagnosis  🗆 Be involved in decisions about their future care  🗆 Make financial arrangements with their family  🗆 Access treatments and support  🗆 Other (please specify) |

**We would like to know more about why you chose this option.**

| **7b. Please select all the reasons that apply from the list below.** |
| --- |
| 🗆 There is no cure / no benefit of them knowing  🗆 Risk of incorrect diagnosis  🗆 It would cause them to feel depressed  🗆 It would make them feel bad about themselves  🗆 Fear that people would treat them differently  🗆 To avoid unnecessary worry  🗆 To avoid putting strain on their relationships  🗆 Fear that knowing the diagnosis might make symptoms progress faster  🗆 So they could live normally for as long as possible  🗆 Other (please specify) |
